# Alternation of Resting-State Functional Connectivity Between Visual Cortex and Hypothalamus in Guinea Pigs With Experimental Glucocorticoid Enhanced Myopia After the Treatment of Electroacupuncture

**DOI:** 10.3389/fninf.2020.579769

**Published:** 2021-01-13

**Authors:** Tao Zhang, Qian Jiang, Furu Xu, Ruixue Zhang, Dezheng Liu, Dadong Guo, Jianfeng Wu, Ying Wen, Xingrong Wang, Wenjun Jiang, Hongsheng Bi

**Affiliations:** ^1^The First College of Clinical Medicine, Shandong University of Traditional Chinese Medicine, Jinan, China; ^2^Department of Ophthalmology and Optometry, Shandong University of Traditional Chinese Medicine, Jinan, China; ^3^Shandong Province Key Laboratory of Integrated Traditional Chinese and Western Medicine for Prevention and Therapy of Ocular Disease, Eye Institute of Shandong University of Traditional Chinese Medicine, Jinan, China; ^4^Affiliated Eye Hospital of Shandong University of Traditional Chinese Medicine, Jinan, China

**Keywords:** functional connectivity, resting-state fMRI, neuroimaging, electroacupuncture, glucocorticoid, myopia

## Abstract

Excessive glucocorticoids (GC) may lead to the aggravation of several basic diseases including myopia, due to plasma hormone imbalances associated with the hypothalamic–pituitary–adrenal axis (HPAA). Electroacupuncture (EA) is an effective therapeutic method to treat many diseases, although it remains unclear whether EA at acupoints on the foot or back would be effective in treating eye diseases. It was recently found that visual cortex activity for responses to visual stimuli with spatial frequency and resting-state functional connectivity (FC) between the supramarginal gyrus and rostrolateral prefrontal cortex was significantly reduced in patients with high myopia. The present study aims to investigate the role of the alternation of resting-state FC among the bilateral visual cortex and hypothalamus in exerting anti-myopia effects of EA in GC-enhanced lens-induced myopic (LIM) guinea pigs such that the mechanisms of EA to treat GC-enhanced myopia at Shenshu (BL23) acupoints can be probed. To confirm the effects of EA, ocular parameters including axial length and GC-associated physiological parameters such as animal appearance, behavior, bodyweight, and levels of four HPAA-associated plasma hormones [free triiodothyronine (FT3), free thyroxine (FT4), estradiol (E2), and testosterone (T)] were also collected. Increased resting-state FC between the left and right visual cortex was detected in GC-enhanced lens-induced myopic guinea pigs with EA at BL23 acupoints (LIM+GC+EA) guinea pigs compared to GC-enhanced lens-induced myopic guinea pigs with EA at sham acupoints (LIM+GC+Sham) guinea pigs, as well as suppressed myopia and recovery of symptoms initially caused by overdose of GC. Recovered symptoms included improved animal appearance, behavior, bodyweight, and HPAA-associated plasma hormone levels were observed after 4 weeks of EA treatment. In contrast, the LIM+GC+Sham group showed decreased FC with elongation of axial length for myopization as compared to the control group and LIM group and exhibited a deterioration in physiological parameters including reduced body weight and balance disruption in the four measured HPAA-associated plasma hormones. Our findings suggest that EA could effectively treat GC-enhanced myopia by increasing resting-state FC between the left and right visual cortices, which may be pivotal to further understanding the application and mechanisms of EA in treating GC-enhanced myopia.

## Introduction

High myopia is a major public health concern, often accompanied by several severe comorbidities, including retinal detachment, cataracts, and glaucoma due to the elongation of axial length (Rudnicka et al., 2016; Morgan et al., 2018). Currently, excessive myopic axial length elongation and increased risk of irreversible visual impairment have been found in experimental lens-induced myopia after intraperitoneal injection of the glucocorticoids (GC) (Ding et al., 2018). The imbalance of four plasma hormones associated with the hypothalamic–pituitary–adrenal axis (HPAA), including free triiodothyronine (FT3), free thyroxine (FT4), estradiol (E2), and testosterone (T), is often caused by excess GC. Excess GC also causes deteriorated physical conditions as well as a reduction in body weight and then resulted in enhancement of basic disorders, such as arthritis and diabetes (De Bosscher and Haegeman, 2009; Lu et al., 2011; Vieira et al., 2011; Wang et al., 2015; Ferreira et al., 2016; Oray et al., 2016; Xia et al., 2017; Yan et al., 2017; Hasona, 2018; Panettieri et al., 2019). Interestingly, electroacupuncture (EA) has been proven to be an effective therapeutic method to treat GC-induced diseases at the Shenshu (BL23) acupoint which was located adjacent to the second lumbar vertebra on the back (Wang et al., 2015; Feng et al., 2018).

Using resting-state functional MRI (rsfMRI), functional connectivity (FC) between brain regions can be assessed by analyzing the temporal relationships of blood oxygen level-dependent (BOLD) fluctuations between brain regions (Chong et al., 2019; O'Neill et al., 2019). In recent years, various studies utilized rsfMRI to investigate the underlying mechanisms of eye diseases including myopia and amblyopia (Hu et al., 2018; Dai et al., 2019). Previous studies reported that visual cortex activity for responses to visual stimuli with spatial frequency, and resting-state FC between the supramarginal gyrus and the rostrolateral prefrontal cortex, was significantly reduced in high-myopia patients (Zhai et al., 2016; Mirzajani et al., 2017). It was also reported that FC density significantly decreased in the posterior cingulate cortex/precuneus (PCC/preCun) (Zhai et al., 2016). However, the role of resting-state FC between the left and right visual cortex on the treatment of high myopia has not yet been explored.

Consequently, there is considerable interest in discovering means to explore whether EA would affect brain function in treating GC-enhanced eye diseases at BL23 acupoints located on the back. In the present study, we aimed to investigate alternation of resting-state FC between the visual cortex and hypothalamus to assess the effects of EA at BL23 acupoints on the treatment of GC enhanced myopia in guinea pigs. We also measured myopia-related ocular parameters including axial length, and GC-associated physiological parameters including animal appearance, behavior, body weight, and levels of four plasma hormones related to the HPAA (FT3, FT4, E2, and T). Our study may provide insights in deepening understanding of the mechanisms of acupuncture in the treatment of GC-enhanced eye diseases at acupoints far from the eyes.

## Materials and Methods

### Animals

Sixty male pigmented guinea pigs (Cavia porcellus) at the age of 2–3 weeks were obtained from the Jinan Xijueling Laboratory Animal Ltd. (Jinan, China) and raised in the animal lab center within the Eye Institute of Shandong University of Traditional Chinese Medicine. Food and water for the guinea pigs were available *ad libitum*, and the room temperature was maintained at 22°C. The guinea pigs were reared in plastic cages (15 cm × 26 cm × 32 cm) under a 12/12 h light–dark cycle. The average light in the cage was ~300 lux. All experimental protocols and animal handling procedures were approved by the ethics committee of the Eye Institute of Shandong University of Traditional Chinese Medicine (2017-002)s and were in accordance with the statement of the Association for Research in Vision and Ophthalmology for the use of animals in vision and ophthalmological research.

### GC Administration and LIM Establishment

The guinea pigs were randomly divided into four groups: control, LIM, LIM+GC+Sham, and LIM+GC+EA. The control group includes animals with no treatment (*n* = 15), and the LIM group comprises of animals with lens-induced unilateral myopization by goggles with a refractive power of −10 diopters glued onto the orbital rim of right eyes (*n* = 15). The LIM+GC+Sham group includes animals with lens-induced unilateral myopization of the right eyes and intraperitoneal injection of hydrocortisone in a dose of 10 mg/kg once daily (8:00–10:00 a.m.) for 2 consecutive weeks and then followed in a dose of 5 mg·kg^−1^ for the next 4 consecutive weeks to maintain the treatment effect, with EA at sham acupoints (*n* = 15), and the LIM+GC+EA group includes animals with lens-induced unilateral myopization of the right eyes and intraperitoneal injection of hydrocortisone in a dose of 10 mg/kg once daily (8:00–10:00 a.m.) for 2 consecutive weeks and then followed in a dose of 5 mg·kg^−1^ for the next 4 consecutive weeks to maintain the treatment effect, with EA at bilateral BL23 acupoints (*n* = 15).

The animals underwent body weight measurement at baseline and at each follow-up examination. The sonographic ocular biometry for axial length measurement was also collected by A/B-mode scan (oscillator frequency: 11 MHz; Quantel Co., Les Ulis, France) at these time points. One drop of 1% cyclopentolate hydrochloride (Alcon, USA) was applied to both eyes to achieve a completely dilated pupil and cycloplegia.

### Electroacupuncture

After the combined treatment of lens-induced myopia and intraperitoneal injection of hydrocortisone for 2 consecutive weeks, the guinea pigs in the LIM+GC+EA group received EA at the bilateral BL23 point for 30 min a day for 4 consecutive weeks. BL23 is located adjacent to the second lumbar vertebra on the back (Xiang et al., 2019). The guinea pigs in the LIM+GC+Sham group were treated with EA at a sham point, which was set to the “degenerated tail” on the gluteus muscle, a point further away from the traditional meridians (Wang et al., 2007). The animals were lightly immobilized using a manufactured apparatus to minimize restraint stress, and acupuncture needles (40 mm in length, 0.30 mm in diameter) were bilaterally inserted to a depth of 8 mm at BL23 once a day (2:00 p.m.). Acupuncture needles were stimulated with an electrical-stimulator (Suzhou Medical Appliance Factory of China, Model SDZ-V), and parameters were set as continuous wave electrical pulses (0.1 ms duration), with a frequency 2 Hz and an intensity of 2 mA.

### Serum Collection and Radioimmunoassay

The blood was drawn by cardiac puncture under anesthesia at 2 p.m. at the 0, 2, and 6 week intervals. Plasma FT3, FT4, E2, and T concentrations were determined in duplicate using standard radioimmunoassay (RIA) techniques by means of 125I-RIA kits with detection limits of 5 × 10–13 M, 1 × 10–12 M, 7.7 × 10–12 M, and 6.6 × 10–11 M, respectively. The different concentrations of the various hormones were measured using a gamma counter (GC-911, Anhui Ustc Zonkia Scientific Instruments Co., Ltd, China). The experimental steps were performed according to the protocols of the kits: (1) 125I FT3 and FT4 RIA kits (North Biotechnology Research Institute, Beijing) and (2) 125I E2 and T RIA kits (Tianjin JiuDing Medicine Bio-Engineering Co., Ltd, Tianjin).

### MRI

Each group included six randomly selected guinea pigs for MRI after 4 weeks of EA. MRI was performed on a BioSpec 70/20 animal MRI system (Bruker BioSpin) equipped with a 7.0-T magnet with a horizontal bore 20 cm in diameter. The operating system was ParaVision 6.0.1, and the maximum gradient strength of the gradient system was 100 mT/m, using a low-temperature phased array receiver coil. During MRI, low-dose isoflurane (Ruiward Life Technology Co., Ltd., Shenzhen, China) was used (3.5% for induction and 1.5% for maintenance), which was slightly adjusted throughout the experiment to maintain a stable breathing frequency of 90 bpm. The animal respiratory rate was monitored using a PC-SAM Small Animal Monitor (SA Instruments). The four groups of guinea pigs were each placed in a separate animal bed equipped with circulating warm water to ensure that body temperature was maintained at 37–38°C through the heated animal bed.

Anatomical images covering the entire guinea pig brain were acquired using a multislice rapid acquisition with a relaxation enhancement sequence with repetition time (TR) = 175 ms, echo time (TE) = 4.5 ms, effective echo time (TE eff) 36 ms, number of averages (NA) = 1, and number of repetitions (NR) = 4, matrix dimension (MD) = 256 ^*^ 256, pixel dimensions (V) = 50 ^*^ 50 mm^2^, slice thickness (STH) = 1 mm, interslice distance (ISD) = 1 mm, and number of slices (NSl) = 30.

For fMRI, gradient-echo echo-planar imaging (EPI) was used with TR = 1,500 ms, TE = 20 ms, number of repetition (NR) = 180, NA = 1, MD = 256^*^ 256, pixel dimensions = 25 ^*^ 220 mm^2^, slice thickness = 1 mm, interslice distance = 1 mm, number of slices = 30.

### MRI Data Analysis

**Figure 1:**
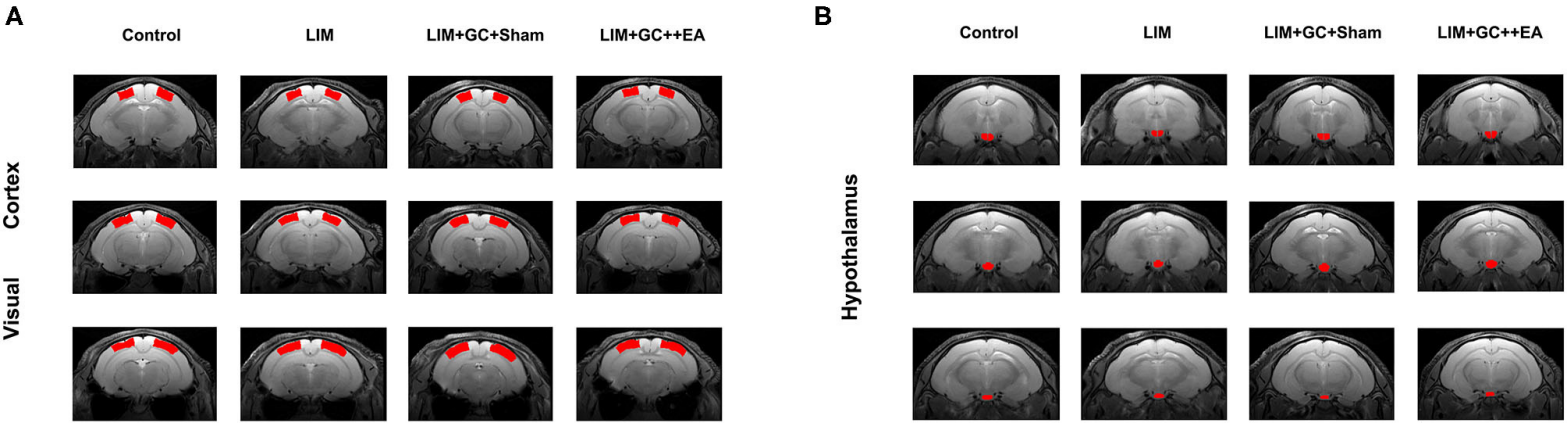
Anatomical locations of the visual cortex ROI **(A)**, based on Paxinos et al., 1980. Anatomical locations of the hypothalamus ROI **(B)**, based on Papp et al., 2014 [control (*n* = 6), LIM (*n* = 5), LIM+GC+Sham (*n* = 4), and LIM+GC+EA (*n* = 6)].

We segmented the label of the visual cortex and hypothalamus manually according to the sixth edition of The Rat Brain in Stereotaxic Coordinates (Figure 1). For a subject's high-resolution T2 anatomical image, we manually performed skull-stripping to remove non-brain tissues. The next step was inhomogeneity correction. Subsequently, a control subject's brain was chosen as the brain template. We registered each subject's T2 volume to the brain template using non-linear registration. Using the transformation generated by the registration algorithm, the subject's visual cortex region (left and right) and the hypothalamus were segmented (Valdes-Hernandez et al., 2011).

The rsfMRI images were realigned and corrected for slice timing. Afterward, a base EPI volume was extracted and skull stripping was performed to remove non-brain tissues. Linear detrending was applied for the removal of a systematic linear trend (Zerbi et al., 2014). The data was then band-pass filtered using a range between 0.01 and 0.3 Hz (Zerbi et al., 2018), and the subject's T2 volume was co-registered to the subject's fMRI base EPI volume. The visual cortex and the hypothalamus in the native fMR space were labeled based on the transformation generated by the registration algorithm. The left and right visual cortex and the hypothalamus were region-of-interests (ROIs), and the mean time courses of these three ROIs were extracted. The pairwise correlation coefficient among the ROIs was calculated to assess the functional connectivity between a brain region pair.

### Statistical Analysis

SPSS software (Version 21.0) was used to perform statistical analyses. All data were expressed as mean ± SEM. An independent sample *t*-test was used to detect differences between groups. Statistical significance was considered when *P* < 0.05.

## Results

### Changes in Morphological Behavior, Body Weight, and Hormone Levels

**Figure 2:**
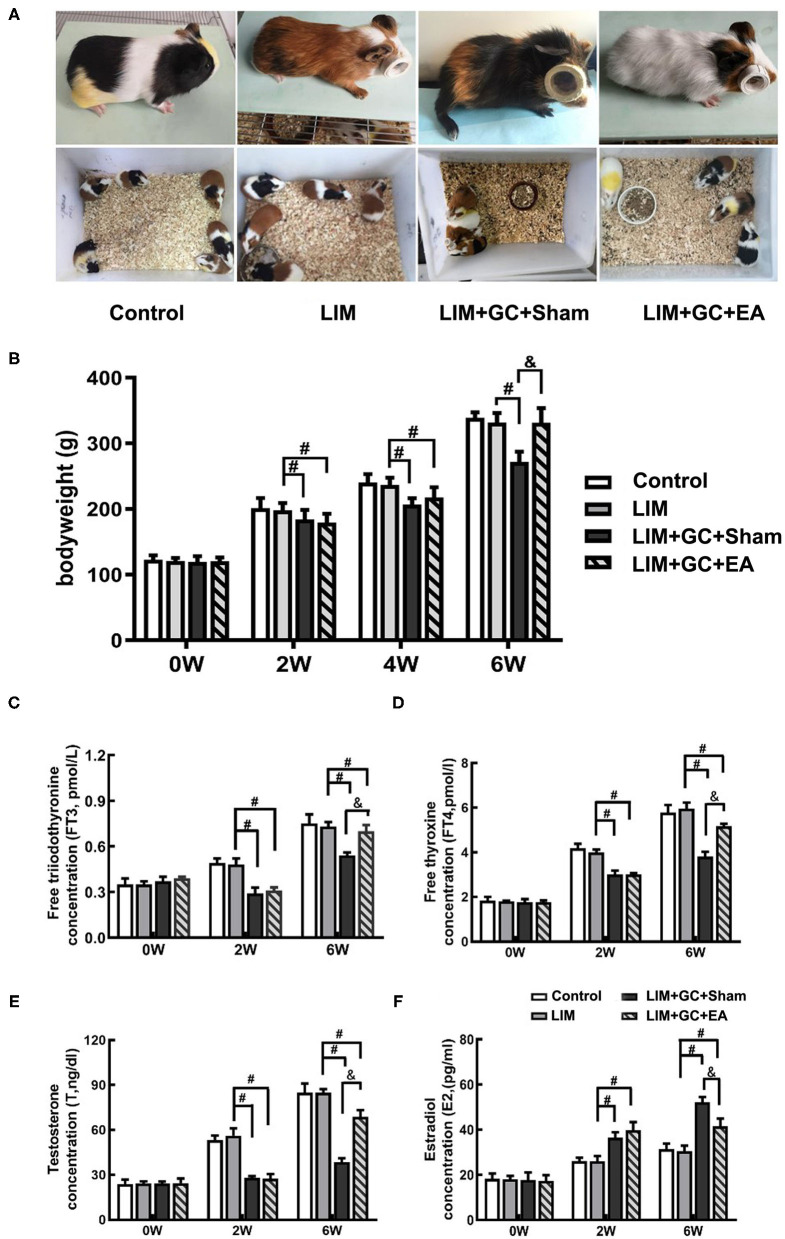
The change in morphological behavior, body weight, and hormone level (*n* = 15 for each group). The changes in morphological behavior after the treatment for 6 w **(A)**, and alterations of body weight **(B)** and hormones, including FT3 **(C)**, FT4 **(D)**, T **(E)**, and E2 **(F)** in serum in all time intervals. ^#^*P* < 0.05 compared with the LIM group; ^&^*P* < 0.05, compared with LIM+GC+Sham group.

At baseline, all groups had similar morphological behavior, body weight, and hormone levels of FT3, FT4, T, and E2 (Figures 2B–F). After 2 weeks of treatment, GC-treated groups (LIM+GC+Sham and LIM+GC+EA) showed deteriorated animal appearance such as dull coats, shivering, and decreased activity when compared to the LIM group and the control group. In addition, the GC-treated groups (LIM+GC+Sham and LIM+GC+EA) showed significantly decreased body weight when compared to the LIM group and the control group (Figure 2B). No significant differences were observed between LIM and controls (Figure 2B). The GC-treated groups (LIM+GC+Sham and LIM+GC+EA) also had significantly decreased concentrations of FT3, FT4, and T and significantly increased concentrations of E2 compared to LIM and control groups (Figures 2C–F).

After 4 weeks of EA treatment (at the time point of “6 weeks,” as EA treatment was conducted after 2 weeks of LIM+GC treatment), the LIM+GC+EA group showed significantly increased body weight compared to the LIM+GC+Sham group, suggesting the GC-induced symptoms were ameliorated by EA (Figures 2A,B). In addition, the LIM+GC+EA guinea pigs also showed significantly increased FT3, FT4, and T concentrations with significantly decreased E2 when compared to the LIM+GC+Sham group (Figures 2C–F).

### Changes of Axial Length

**Figure 3:**
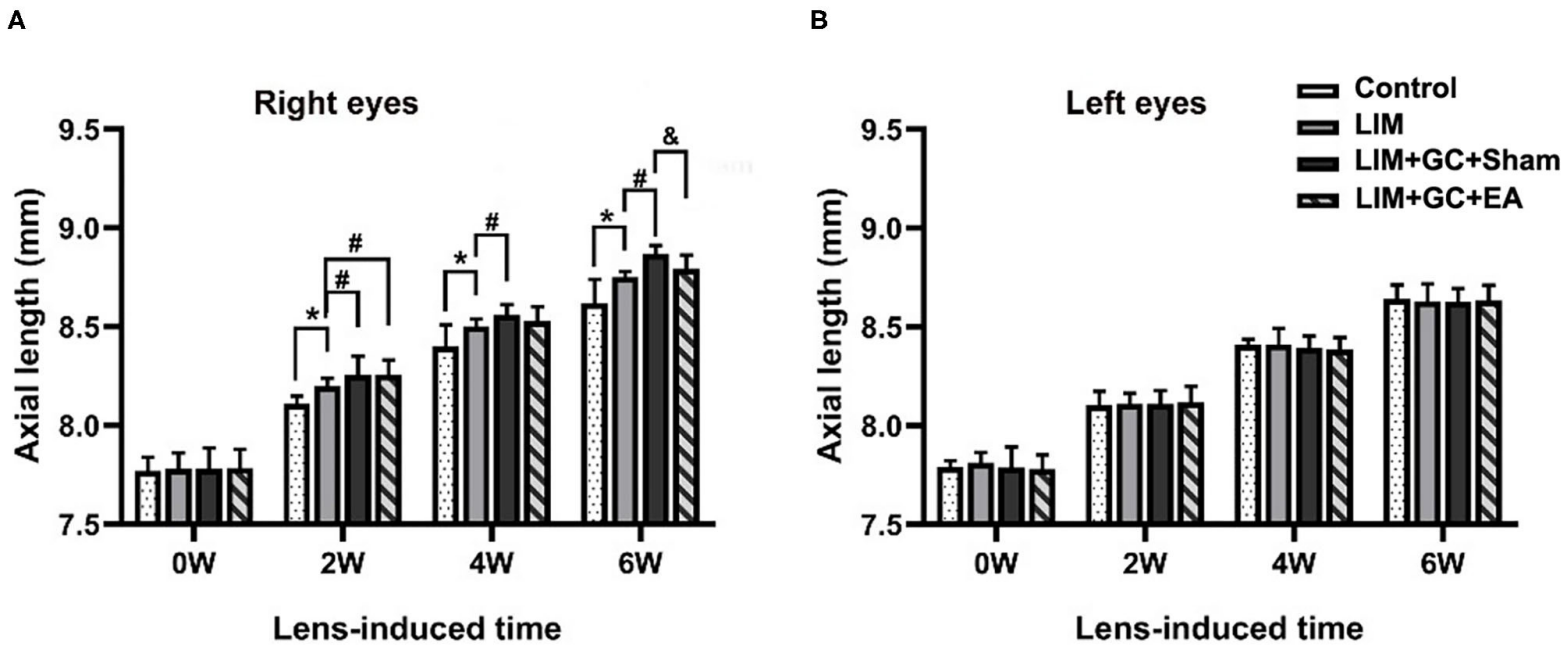
Changes in axial length at various intervals (*n* = 15 for each group). The changes in axial length of right eyes **(A)** and left eyes **(B)** in control, LIM, LIM+GC+Sham, and LIM+GC+EA group at the 0, 2, 4, and 6 week interval (*n* = 15 per group). *P < 0.05 compared with the control group; ^#^*P* < 0.05 compared with the LIM group; ^&^*P* < 0.05, compared with LIM+GC+Sham group.

At baseline, a similar mean axial length of right eyes (treated) and left eyes (untreated) was observed in all groups (Table 1, Figure 3). In the control group, mean axial length of right (treated) eyes increased from 7.77 ± 0.07 mm (mean ± standard deviation) at baseline to 8.62 ± 0.12 mm at the end of follow-up, and mean axial length of left (untreated) eyes increased from 7.79 ± 0.03 at baseline to 8.64 ± 0.07 mm at the end of follow-up. There was no significant difference between treated and untreated eyes (Table 1). In the LIM group, the mean axial length of right eyes (treated) was significantly longer after 2 weeks of treatment compared to the control group (LIM group vs. control group, 8.20 ± 0.04 mm vs. 8.11 ± 0.04 mm, *P* < 0.001) and this increase continued in a time-dependent manner (Table 1, Figure 3A).

**Table 1:** Sonographic biometric measurements (mean ± standard deviation; OD: right eyes; OS: right eyes).

**Group**	**Time (week)**	**OD (mm)**				**OS (mm)**			
		**Vitreous cavity length**	**Anterior chamber depth**	**Lens thickness**	**Axial length**	**Vitreous cavity length**	**Anterior chamber depth**	**Lens thickness**	**Axial length**
Control	0	3.42 ± 0.05	1.16 ± 0.01	3.18 ± 0.05	7.77 ± 0.07	3.43 ± 0.06	1.17 ± 0.02	3.19 ± 0.02	7.79 ± 0.03
	2	3.49 ± 0.03	1.22 ± 0.03	3.40 ± 0.02	8.11 ± 0.04	3.50 ± 0.05	1.20 ± 0.03	3.40 ± 0.04	8.10 ± 0.07
	4	3.58 ± 0.09	1.23 ± 0.02	3.60 ± 0.05	8.40 ± 0.11	3.59 ± 0.04	1.23 ± 0.01	3.59 ± 0.04	8.41 ± 0.03
	6	3.63 ± 0.07	1.26 ± 0.02	3.73 ± 0.06	8.62 ± 0.12	3.67 ± 0.06	1.25 ± 0.03	3.72 ± 0.04	8.64 ± 0.07
LIM	0	3.42 ± 0.05	1.16 ± 0.03	3.18 ± 0.04	7.78 ± 0.08	3.44 ± 0.04	1.17 ± 0.02	3.20 ± 0.04	7.81 ± 0.05
	2	3.55 ± 0.04^*^	1.21 ± 0.04	3.45 ± 0.03	8.20 ± 0.04^*^	3.50 ± 0.05	1.20 ± 0.03	3.41 ± 0.03	8.11 ± 0.05
	4	3.65 ± 0.03^*^	1.22 ± 0.03	3.63 ± 0.04	8.50 ± 0.04^*^	3.57 ± 0.06	1.23 ± 0.02	3.61 ± 0.03	8.41 ± 0.08
	6	3.74 ± 0.02^*^	1.26 ± 0.02	3.75 ± 0.03	8.75 ± 0.03^*^	3.65 ± 0.09	1.25 ± 0.03	3.73 ± 0.03	8.63 ± 0.09
LIM+GC+Sham	0	3.43 ± 0.08	1.16 ± 0.03	3.18 ± 0.07	7.78 ± 0.10	3.43 ± 0.05	1.16 ± 0.03	3.20 ± 0.07	7.79 ± 0.10
	2	3.57 ± 0.04^*^, ^#^	1.21 ± 0.02	3.47 ± 0.08^*^	8.26 ± 0.10^*^	3.50 ± 0.04	1.21 ± 0.03	3.41 ± 0.04	8.11 ± 0.07
	4	3.69 ± 0.03^^*^^,^^#^^	1.24 ± 0.02	3.64 ± 0.05	8.56 ± 0.05^*^	3.57 ± 0.03	1.24 ± 0.02	3.59 ± 0.04	8.40 ± 0.06
	6	3.85 ± 0.04^*^^,^ ^#^	1.26 ± 0.02	3.77 ± 0.03	8.87 ± 0.04^*^, *^#^*	3.64 ± 0.05	1.26 ± 0.02	3.73 ± 0.06	8.63 ± 0.07
LIM+GC+EA	0	3.43 ± 0.06	1.16 ± 0.03	3.19 ± 0.05	7.78 ± 0.10	3.42 ± 0.05	1.16 ± 0.03	3.20 ± 0.05	7.78 ± 0.07
	2	3.57 ± 0.04^*^^,^^#^	1.20 ± 0.02	3.48 ± 0.06^*^	8.26 ± 0.07^*^	3.52 ± 0.06	1.19 ± 0.02	3.41 ± 0.06	8.12 ± 0.08
	4	3.68 ± 0.03^*^	1.23 ± 0.02	3.63 ± 0.04	8.54 ± 0.03^*^	3.56 ± 0.06	1.23 ± 0.02	3.60 ± 0.06	8.39 ± 0.06
	6	3.79 ± 0.05^*^^,^^&^	1.26 ± 0.04	3.74 ± 0.05	8.79 ± 0.07^*^^,^^&^	3.66 ± 0.08	1.25 ± 0.03	3.73 ± 0.06	8.63 ± 0.08

* P < 0.05 compared with the control group;

# P < 0.05 compared with the LIM group;

& *P < 0.05, compared with the LIM+GC+Sham group (n = 15)*.

In groups with unilateral lens-induced myopization and intraperitoneal injection of hydrocortisone (LIM+GC+Sham and LIM+GC+EA), the mean axial lengths of the right eyes (treated eyes) were significantly longer compared to the LIM group after combined treatment of GC and lens-induced myopization for 2 weeks, thus suggesting that excess GC significantly increased the degree of lens-induced myopia [(LIM+GC+Sham group vs. LIM group, 8.26 ± 0.10 mm vs. 8.20 ± 0.04 mm, *P* < 0.05; LIM+GC+EA group vs. LIM group, 8.26 ± 0.07 mm vs. 8.20 ± 0.04 mm, *P* < 0.05] (Table 1, Figure 3A). However, there was no significant difference in axial length between LIM+GC+Sham and LIM+GC+EA at that time point (LIM+GC+EA vs. LIM+GC+Sham, 8.26 ± 0.10 mm vs. 8.26 ± 0.07 mm, *P* = 0.833) (Table 1, Figure 3A).

Interestingly, after the treatment of EA at BL23 for 4 weeks, the axial length of the right eyes was significantly shorter in the LIM+GC+EA group compared to the LIM+GC+Sham group (LIM+GC+EA vs. LIM+GC+Sham, 8.79 ± 0.07 mm vs. 8.87 ± 0.04 mm, *P* < 0.05) (Table 1, Figure 3A). No significant difference was found in the axial lengths of the left eyes (untreated eyes) among each group at any time interval (Table 1, Figure 3B).

### Altered Resting-State FC

**Figure 4:**
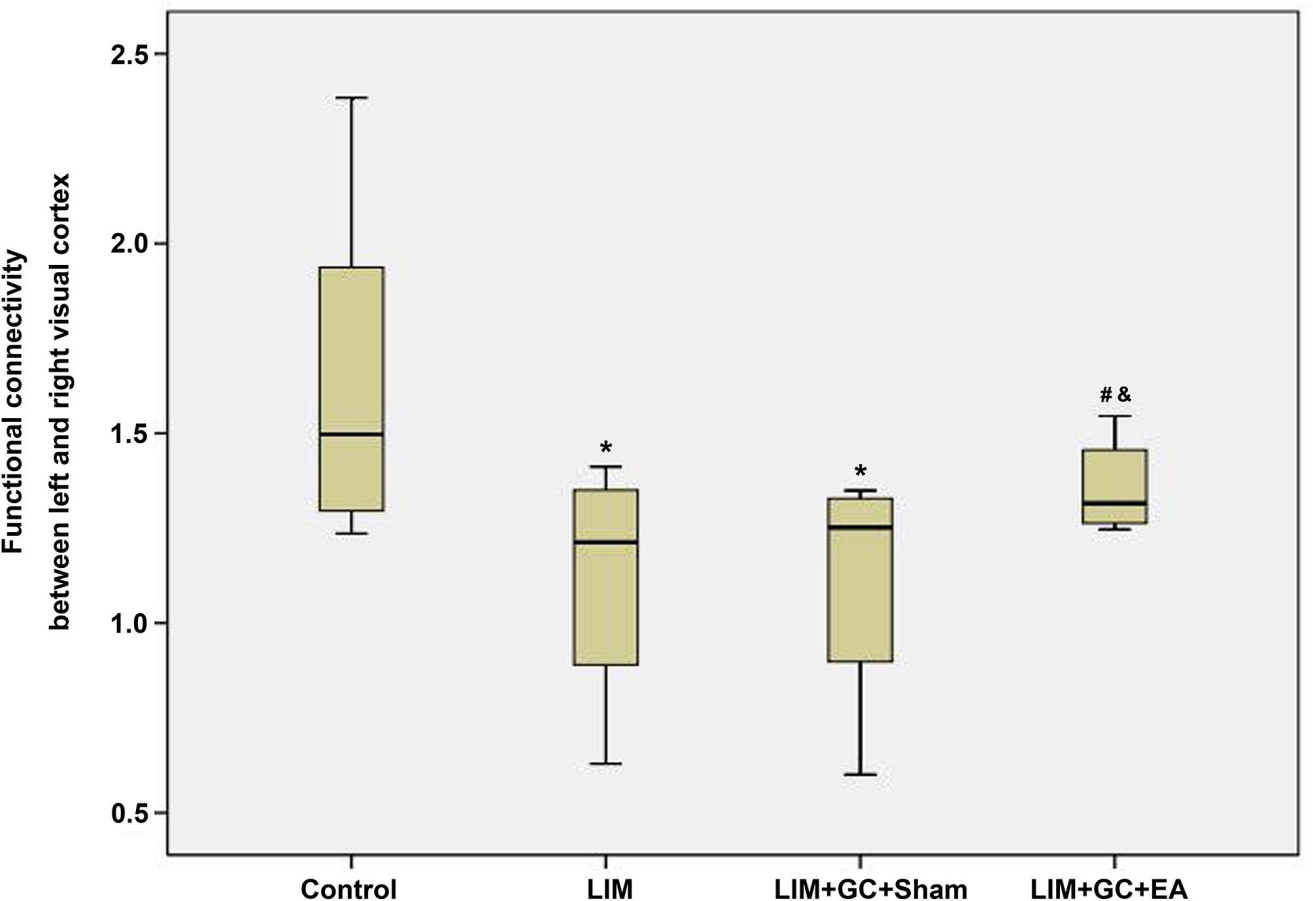
The boxplot of functional connectivity in the visual cortex; *compared with the control group, *P* < 0.05; ^#^compared with the LIM group, *P* < 0.05; ^&^compared with LIM+GC+Sham group, *P* < 0.05 [control (*n* = 6), LIM (*n* = 5), LIM+GC+Sham (*n* = 4), and LIM+GC+EA (*n* = 6)].

Among the four groups, control (*n* = 6), LIM (*n* = 5), LIM+GC+Sham (*n* = 4), and LIM+GC+EA (*n* = 6), there were significant differences across the groups, as shown through linear regression modeling (*p* = 0.05). As shown in Figure 4, we found that the FC between the left and right visual cortices of the LIM group was significantly lower than that of the control (*p* = 0.02). The FC of the visual cortex of the LIM+GC+Sham group was also lower than that of control (*p* = 0.03). Of note, the FC of visual cortex of the LIM+GC+EA group is higher than that of LIM and LIM+GC+Sham groups. The difference in FC between the visual cortices was not significant between control and LIM+GC+EA; no significant difference between the LIM group and the LIM+GC+Sham group was found either (*p* > 0.05). Meanwhile, we did not find any significant difference for FC between the hypothalamus and each side of the visual cortex among all the four groups.

**Figure 5:**
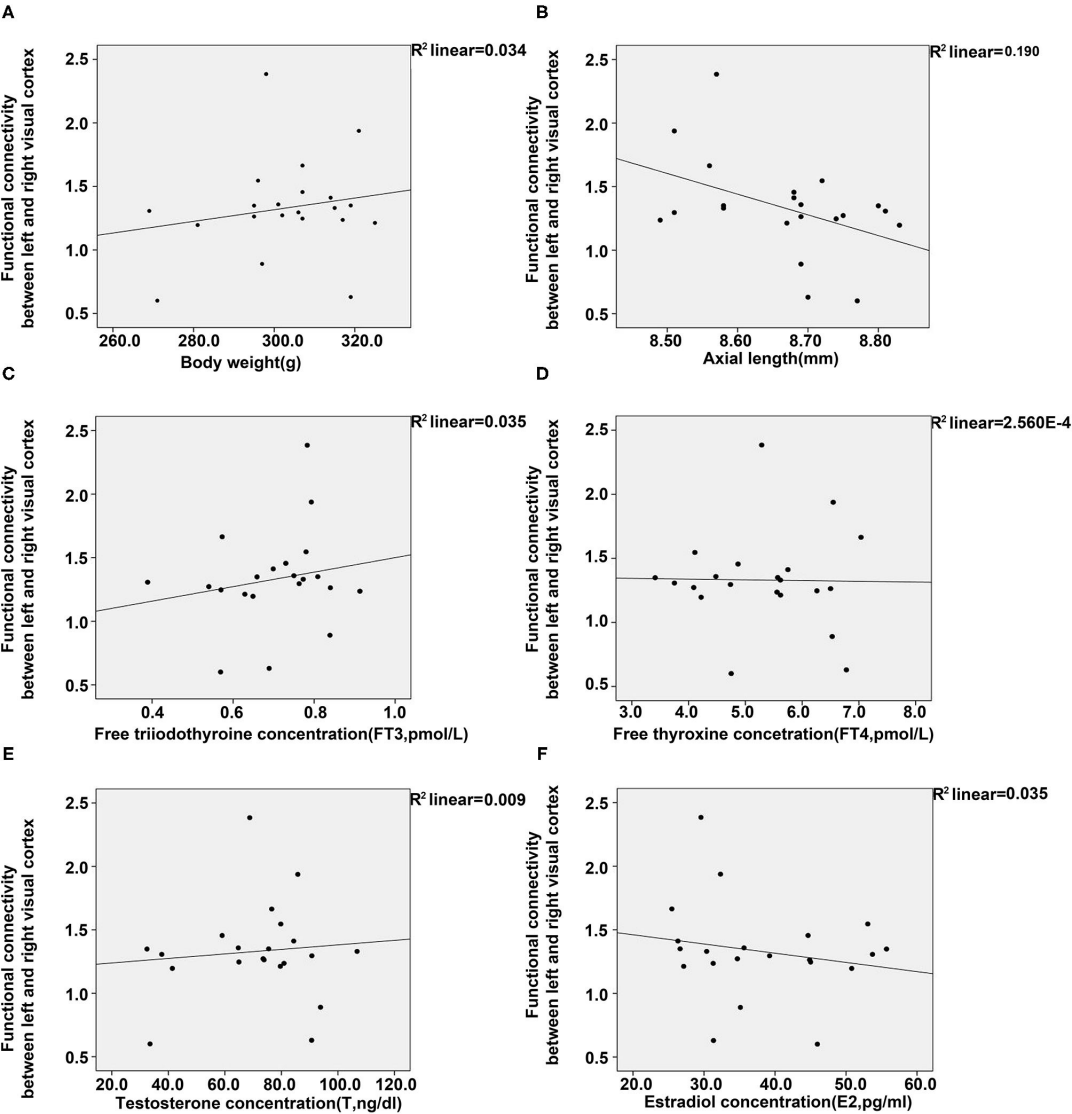
Regression analyses between the functional connectivity in the visual cortex, and body weight, axial length, and four plasma hormones (FT3, FT4, T, E2). **(A)** Regression analyses between the functional connectivity in the visual cortex and body weight **(A)** and axial length **(B)**. **(C–F)** Regression analyses between the functional connectivity in the visual cortex and the concentrations of FT3 **(C)**, FT4 **(D)**, T **(E)**, and E2 **(F)** [control (*n* = 6), LIM (*n* = 5), LIM+GC+Sham (*n* = 4), and LIM+GC+EA (*n* = 6)].

To further elucidate the relationship of FC between the visual cortex and parameters of animals, we conducted regression analyses between them. Results from the analyses showed a positive association between FC of the visual cortex and bodyweight, FT3 and T, whereas a negative association was found between FC of the visual cortex and E2 or axial length (Figure 5). However, no significant relationship was found between FC of the visual cortex and FT4.

## Discussion

The prevalence of myopia has markedly increased within the past three decades, especially in China. The main characteristic of myopia is the irreversible elongation of axial length, resulting in many severe visual impairments including myopic macular degeneration, glaucoma, and even blind (Rudnicka et al., 2016; Morgan et al., 2018). In addition, overdose of GC could also induce excessive myopic axial length elongation and imbalance of four plasma hormones associated with hypothalamic–pituitary–adrenal axis (HPAA), including E2, T, FT3, and FT4 (Ding et al., 2018). The receptors of E2 and T are widely expressed in various ocular tissues (Wickham et al., 1998, 2000; Suzuki et al., 2001).

The guinea pigs have been a classic model to evaluate the effects of various therapeutic methods on myopia as well as its complications. Similar to the process of eye development in human beings, guinea pigs show hyperopia at birth and then rapidly become emmetropia within the first 3 weeks (21 days) of age (Shan et al., 2018). Their eyes have a more similar structure and biometric changes in the development of myopia as human beings, compared to other experimental model animals such as chicks and mice as well (Wu et al., 2020). In the present study, a low dose of isoflurane was used to maintain stable breathing frequency in anesthetized animals, and low-dose isoflurane maintained the resting-state networks of anesthetized animals to be similar to wake ones in rats and mice. The protection in resting-state neuron activity may result from a moderate systemic vasodilator effect due to increasing resting blood flow from low-dose isoflurane (Iida et al., 1998; Guilfoyle et al., 2013; Zhou et al., 2014). Moreover, isoflurane was not reported to be a risk factor for GC signaling or myopiazation, suggesting that it would be suitable for the application in the present study. Due to the relative ease in signal acquisition and proficiency of rsfMRI technology to measure the functional connectivity (FC) between functional areas of the brain in different populations, we choose the axial length and the resting-state functional connectivity using rsfMRI technology to assess the effects of EA at BL23 on GC-enhanced myopia. It strongly suggested that FC between the visual cortices played an important role in myopization.

### FC in the Myopic Guinea Pig's Brain

It has been demonstrated that the amplitude of low-frequency fluctuation values in high-myopia patients are reduced in the right cerebellum anterior lobe/calcarine/bilateral parahippocampal gyrus, bilateral posterior cingulate cortex, and bilateral middle cingulate cortex, while they are significantly increased in the left optic radiation, bilateral frontal parietal cortex, and left primary motor cortex (M1)/primary somatosensory cortex (S1) (Huang et al., 2016; Cheng et al., 2020). Meanwhile, it was found that the high myopia exhibited significantly decreased short- and long-range FC densities in the posterior cingulate cortex/precuneus (PCC/preCun), with a similar result reported in amblyopia patients, which showed decreased FC between the PCC/preCun and bilateral primary visual areas (Ding et al., 2013; Zhai et al., 2016).

Our results indicated that FC between the visual cortex in the LIM+GC+Sham group and LIM group was significantly lower compared to the control group with the elongation of axial length. It exhibited many similarities with previous studies. It was reported that expression of neurotransmitters and their receptors changed in the primary visual cortex during the development of myopia (Zhao et al., 2017). Researches found that induced high myopia caused a significant reduction in the visual cortex activity by presenting a high range of spatial frequencies using functional MRI compared to the normal vision state (Mirzajani et al., 2017). Meanwhile, a resting-state functional magnetic resonance imaging study also demonstrated that low/moderate myopia and high myopia will lead to decreased neuronal and physiological activities in the primary visual cortex by studying the amplitude of low-frequency fluctuations (Cheng et al., 2020).

### Effect of Excess GC on HPAA and Myopia

Evidence has shown that excessive GC resulted in deterioration of various diseases including arthritis and myopia through affecting secretion of certain hormones including FT3, FT4, E2, and T secreted from the hypothalamic pituitary target gland (adrenal, thyroid, and gonad) axis into target gland (Yang et al., 2008; Pace et al., 2009; Ding et al., 2018). It was found that concentrations of FT3, FT4, and T decreased while E2 elevated after the treatment of GC in the present study. These findings are consistent with previous studies that intraperitoneal injection of hydrocortisone, a type of GC, results in deteriorated animal appearance and a reduced body weight, accompanied with the suppression of the HPAA function by affecting the HPAA plasma hormone expression (Yang et al., 2008; Zhao et al., 2013, 2016). Besides, E2, as a member of estrogen, was reported to be a modulating factor that maintains the biomechanical properties and stability of the cornea and upregulate MMP-2 activity and protein expression in human retinal pigment epithelium cells, whereas T acted as an androgen of the steroid family and was reported to be associated with the biochemical characteristics of the sclera, the aqueous outflow pathway, and the iris/ciliary body (Knepper et al., 1985; Marin-Castano et al., 2003; Song et al., 2014). Also, cortisol administration elevated both the default mode network and salience network activity to a normal level to treat traumatic stress disorder and anxiety (Soravia et al., 2018). Also, it was found that intraperitoneal injection of GC can enhance myopic shift and axial elongation in guinea pigs with lens-induced myopia (Ding et al., 2018). These symptoms were also defined as “kidney-yang deficiency” in traditional Chinese medicine, which could be effectively treated by EA (Shen, 1999).

Previous studies showed that rapid intravenous infusion of hydrocortisone significantly increased the fMRI BOLD signal within the hippocampus in a time-dependent manner (Symonds et al., 2012). Additionally, it was also found that increased endogenous GC can elevate the resting-state FC of brain regions highly expressing GC receptors, such as the medial prefrontal cortex and medial temporal lobe (Stomby et al., 2019). Nevertheless, only FC between visual cortices was found to be significantly associated with alteration of levels of HPAA-associated hormones including FT3, T, and E2 in the serum, instead of FC between the hypothalamus and each side of the visual cortex. It might also be suggested that neuron signals between the hypothalamus and visual cortex may be through some unknown intermediate medium. Moreover, FT3 rather than FT4 played an important role in the relationship between GC and FC of the visual cortex.

### Effects of EA on the Brain

Increasing evidence demonstrates that acupuncture at acupoints located in the body such as the limbs and trunk could effectively treat nervous system diseases including stroke, migraines, motor system diseases, and other diseases such as functional dyspepsia hypertension, overweight and Crohn's disease (Cai et al., 2018). It was reported that acupuncture could effectively enhance the FC between left primary motor area and left inferior frontal gyrus to promote the compensatory response to treat refractory facial paralysis, and enhance the functional connectivity between the precentral gyrus and the hippocampus in the Alzheimer disease patients (Zheng et al., 2018; Ma et al., 2019). Acupuncture could effectively enhance the FC between the left primary motor area and left inferior frontal gyrus to promote the compensatory response, increase connectivity between the periaqueductal gray, anterior cingulate cortex, left posterior cingulate cortex, right anterior insula, limbic/paralimbic, and precuneus, and adjust the limbic-paralimbic-neocortical network, brainstem, cerebellum, and subcortical and hippocampus brain areas (Cai et al., 2018).

Researches have shown that EA at acupoints located near the eyes, including Hegu (LI4) and Taiyang (EX-HN5), was effective to improve myopia by downregulating the level of retinal GABA in a myopic guinea pig model (Sha et al., 2015). It was also found that the stimulation of acupoints in the body, such as LR3 located on the feet, activated some areas of the visual cortex (Liu et al., 2012). With the development of rsfMRI technology, more research has focused on exploring different mechanisms of treatment including acupuncture for eye diseases such as high myopia, amblyopia, and blindness, by measuring FC between brain regions (Huang et al., 2016; Mendola et al., 2018; Wen et al., 2018). It was well established that the development of myopia is highly associated with alteration of function of visual cortex (Mirzajani et al., 2017). In the present study, our data revealed that EA at BL23 acupoints, located adjacent to the second lumbar vertebra on the back, could effectively suppress the elongation of axial length induced by a combination of treatment of GC and negative lens through recovering the balance of HPAA-associated plasma hormones and effectively recovering FC between the visual cortex of LIM+GC animals to normal levels, providing strong support to the notion that FC is related to the mechanism of acupuncture. It was consistent with the previous studies that acupuncture at a group of acupoints including BL23 as one of the major acupoints could relieve the symptoms of many disorders by improving the cerebral hemodynamics and cognitive deficits in the hippocampal CA1 region and rebalancing HPA-associated plasma hormones including E2, T, CORT, LH, and GnRH, or inhibiting the expression of orexin in the lateral hypothalamus (Wang et al., 2017, 2020; Zhang et al., 2017; Ji et al., 2019; Jing et al., 2020). Despite the limited research conducted on elucidating the effects of stimulating the BL23 acupoint as a single point to cure Kidney Yang deficiency-associated disorders, it was once reported that acupuncture at BL23 could effectively treat senescence-accelerated mice by increasing levels of serum hormone T (Zhang et al., 2009). These results would provide further evidence for the hypothesis that acupuncture could treat visual impairments including high myopia, through the alteration of the function of the visual cortex at acupoints located far from the eyes.

Nevertheless, several limitations of the study should be mentioned. First, there have been no brain atlases on guinea pigs so far. We therefore performed the MRI data analysis according to the rat brain atlas. Second, the fMRI image in this study was relatively low, so a higher image resolution would definitely strengthen our conclusion. Third, the specific mechanism of glucocorticoids aggravating the development of myopia needs further exploration.

In summary, EA could effectively treat GC-enhanced myopia by increasing resting-state FC between the left and right visual cortices at BL23, which may be pivotal in understanding the underlying mechanisms of EA in the treatment of GC-enhanced myopia.

## Data Availability Statement

The original contributions presented in the study are included in the article/supplementary material, further inquiries can be directed to the corresponding author/s.

## Ethics Statement

The animal study was reviewed and approved by the Ethics Committee of Eye Institute of Shandong University of Traditional Chinese Medicine.

## Author Contributions

HB and WJ conceived and formulated the research. TZ, QJ, FX, and RZ raised animals and performed treatment. WJ, QJ, FX, DL, and DG conducted rsfMRI measurement. WJ and QJ analyzed the data. WJ, QJ, and TZ wrote the paper. All authors read and approved the final manuscript.

## Conflict of Interest

The authors declare that the research was conducted in the absence of any commercial or financial relationships that could be construed as a potential conflict of interest.
